# Pomegranate peel polyphenols inhibits inflammation in LPS-induced RAW264.7 macrophages via the suppression of TLR4/NF-κB pathway activation

**DOI:** 10.29219/fnr.v63.3392

**Published:** 2019-04-23

**Authors:** Lin Du, Jianke Li, Xitong Zhang, Lifang Wang, Weimin Zhang, Mi Yang, Chen Hou

**Affiliations:** 1 Department of Food Quality and Safety, College of Food Engineering and Nutritional Science, Shaanxi Normal University, Xi’an, China; 2 Department of Food Quality and Safety, College of Food & Bioengineering, Henan University of Science and Technology, Luoyang, China; 3 University Key Laboratory of Food Processing Byproducts for Advanced Development and High Value Utilization, Shaanxi Normal University, Xi’an, China; 4 National Engineering Laboratory for Resource Development of Endangered Crude Drugs in Northwest China, Shaanxi Normal University, Xi’an, China

**Keywords:** pomegranate peel polyphenols, punicalagin, ellagic acid, anti-inflammation, toll-like receptors, nuclear factor-kappa B, cell signaling pathway

## Abstract

**Backgrounds:**

Inflammatory response mediated by activated immune cells is a vital process in host defense system while responding to various stresses. Our previous studies have indicated that pomegranate peel polyphenols (PPPs) and their main components punicalagin (PC) and ellagic acid (EA) decreased pro-inflammatory cytokines and inflammatory mediators by regulating the mitogen-activated protein kinases (MAPKs) pathway, but whether these tested polyphenols play an important role in NF-κB signaling pathway, another crucial pathway of inflammation, remains unclear.

**Objective:**

In this study, we analyzed the anti-inflammatory effect of these polyphenols via TLR4-NF-κB pathway in lipopolysaccharide (LPS)-induced RAW264.7 macrophages.

**Methods:**

Different concentrations of PPPs, PC, and EA were pre-incubated with RAW264.7 macrophages and then stimulated with LPS (1 μg/mL), and the effects of reactive oxygen species and TLR4 were investigated. Moreover, NF-κB p65 nuclear translocation and phosphorylation, and degradation of IκB were measured by Western blot. Furthermore, the influence of pro-inflammatory cytokines was detected by enzyme-linked immunosorbent assay (ELISA).

**Results:**

Our data showed that PPPs, PC, and EA inhibited LPS-induced intracellular ROS production and suppressed the mRNA and protein expression levels of TLR4 in a dose-dependent manner. Moreover, the anti-inflammatory mechanism was involved in blocking LPS-induced phosphorylation, degradation of IκB, and nuclear translocation of p65. Additionally, PPPs and PC exhibited a stronger anti-inflammatory effect than that of EA.

**Conclusion:**

The results indicated that PPPs possess potent anti-inflammatory effect, and PC was the main effective component in PPPs, which provided new insights into the utilization of PPPs to prevent inflammation-associated disorders.

## Popular scientific summary

- PPPs, PC and EA have obvious anti-inflammatory effect.
- PPPs, PC and EA can inhibit ROS production and TLR4 expression in LPS-induced RAW264.7 macrophages.
- The molecular mechanism is associated with the inhibiting the activation of NF-κB pathway, by blocking LPS-induced phosphorylation and degradation of IκBα, and subsequently, p65 nuclear translocation.
- The anti-inflammatory effect of PC was much better than EA, and PC was the main effective component among PPPs in anti-inflammation.

Inflammatory response plays an important role in both normal physiology and pathology (1). Prolonged inflammation can lead to many chronic diseases, including diabetes, cardiovascular disease, cancer, arthritis, and neurodegenerative diseases (2). The activation of inflammatory cascade produces various inflammatory mediators, such as nitric oxide (NO), prostaglandin E2 (PGE_2_), and pro-inflammatory cytokines including TNF-α, IL-1β, and IL-6 (3).

The production of reactive oxygen species (ROS) by phagocytic leukocytes (neutrophils, monocytes, macrophages, and eosinophils) is one of the most important characteristics in the inflammatory process. ROS activation could act as a significant and adverse participant in abnormal inflammatory diseases, and studies have shown that various antioxidants are able to prevent NF-κB activation (4, 5) and intracellular ROS generation (6).

The nuclear factor-kappa B (NF-κB) pathway has been identified as a key mediator of inflammation and serves as an important target for drug development (7). In normal cells, inactivated NF-κB is bound to inhibitor of κB (IκB) in the cytoplasm. In terms of cell stimulation, NF-κB is activated by the phosphorylation of IκB, which further leads to ubiquitination and proteasome degradation of IκB (8). The resulted free NF-κB translocates to the nucleus, where it binds to κB-binding sites in the promoter regions of target genes and induces the transcription of pro-inflammatory mediators and cytokines (9, 10).

The pomegranate (*Punica granatum* L.) originated from the Middle East is now widely cultivated and consumed throughout the world. Currently, as a kind of polyphenol-rich fruit, it has potentially anti-inflammatory, anti-diabetic, anti-microbial, anti-obesity, anti-oxidative anti-cancer, and hypolipidemic effect *in vivo* and *in vitro* (11–17). PC is a unique and major polyphenolic component of pomegranate fruits, especially rich in the peel (18), which can release EA upon hydrolysis (19). Our laboratory analysis has shown that PPPs, PC, and EA have remarkable contributions in anti-oxidant (20, 21), anti-cancer (22), inhibition lipid accumulation, and enhancement cholesterol efflux (23–25). Our previous studies have shown that PPPs and their main components PC and EA exert significant anti-inflammatory effects, which reduced the pro-inflammatory cytokines release by inhibiting MAPK activation. In addition, toll-like receptors (TLRs) play a crucial role in the molecular mechanisms of inflammatory processes (26). TLR4, a pattern-recognition receptor (PRR), emerges as a key player in the initiation and activation of inflammation and innate immune responses, leading to intracellular signaling cascade initiation. The study has shown that TLR4 signaling is responsible for MAPKs activation and NF-κB translocation (27). Thus, in order to further clarify the specific mechanisms of PPPs on regulating inflammatory response, and to further clarify the different efficacies among these pomegranate active components, we applied our current research.

## Materials and methods

### Chemicals

Pomegranate fruits were obtained from Lintong, Shaanxi province of China, and the extraction and purification of PPPs were performed in our laboratory (20, 21). Detailed methods and results have been presented in the previous article (28). The polyphenol content of PPPs was 57.09%. The main polyphenol compounds were gallic acid, punicalagin (punicalagin-α and punicalagin-β), catechin, chlorogenic acid, epicatechin, and ellagic acid. The yielding of PC, which is a major component of PPPs, was 464.48 mg/g, and the yielding of EA was 71.50 mg/g. The concentration of the other components of PPPs – catechin, gallic acid, epicatechin, and chlorogenic acid – was 45.14, 38.24, 35.28, and 8.85 mg/g, respectively.

Punicalagin, ellagic acid, and LPS (*Escherichia coli* 055:B5) were purchased from Sigma-Aldrich (St. Louis, MO, USA). Cell culture reagents and fetal bovine serum were purchased from Gibco BRL (Rockville, MD, USA). TAK-242 was purchased from MedChemExpress (Monmouth Junction, NJ, USA). Bay11-7082, MG-132, ROS, and NO assay kits were purchased from Beyotime Biotechnology (Beijing, China). TLR4, P65, *p*-IκBα, IκBα, histone 1, and β-actin primary antibodies for Western blot analysis were purchased from Cell Signaling Technology, Inc. (Beverly, MA, USA).

### Cell culture

Murine macrophage cell line RAW264.7 was obtained from the Cell Bank of the Chinese Academy of Sciences (Shanghai, China) and was cultured in DMEM supplemented with 10% FBS, 1% streptomycin/penicillin at 37 °C in a 5% CO_2_ incubator. The cells were sub-cultured every 2 days at 1:3 split ratios.

### Measurement of intracellular ROS

Intracellular ROS release was measured by detecting the fluorescence intensity of the oxidant-sensitive probe 2’, 7’-dichlorfluorescein-diacetate (DCFH-DA). RAW264.7 macrophages (3 × 10^5^ cells/well in 12-well culture plate) were incubated with different concentrations of PC (1, 10, and 50 μM), EA (1, 10, and 50 μM), and PPPs (1, 10, and 100 μg/mL) for 1 h before exposure to LPS (1 μg/mL) for 24 h at 37 °C (5% CO_2_). Then, with additional 10 μM DCFH-DA, they were incubated at 37 °C for 25 min. The cells were washed three times with PBS, and then one part of the cells was evaluated by using a fluorescence microscope (Leica DMIL LED, Leica, Germany), and another part of the cells was harvested and detected by a fluorescence microplate reader with excitation at 488 nm and emission at 525 nm.

### Reverse transcription and quantitative real-time PCR

RAW264.7 macrophages were plated at 4 × 10^5^ cells/well in 6-well plates and incubated overnight. The cells were then treated with the tested polyphenols or vehicle [0.2% dimethyl sulfoxide (DMSO) for 1 h prior to the stimulation with LPS (1 μg/mL)]. After 24 h of LPS treatment, the cells were collected, and the total RNA was extracted by Total RNA Kit I (Omega, Norcross, GA, USA). Reverse transcription was performed with 1 μg of total RNA using Revert Aid TM First Strand cDNA Synthesis Kit (Thermo, Boston, MA, USA). Real-time Polymerase Chain Reaction (PCR) was performed using Maxima SYBR Green/Fluorescein qPCR Master Mix (Thermo, Boston, MA, USA). The PCR primers were synthesized by Sangon Biotech Co., Ltd (Shanghai, China), and the primer sequences are listed in Table 1.

**Table 1:** Primer information

Primer name	Forward (5’-3’)	Reverse (5’-3’)	Size (bp)
TLR4	GGC CAA TTT TGT CTC CAC AG	GCA TGG CTT ACA CCA CCT CT	124
NF-κB	ATG TAG TTG CCA CGC ACA GA	GGG GAC AGC GAC ACC TTT TA	181
β-actin	GTG CTA TGT TGC TCT AGA CTT CG	ATG CCA CAG GAT TCC ATA CC	174

### Nitric oxide determination

RAW264.7 macrophages were plated at 4 × 10^5^ cells/well in 6-well plates and pre-incubated with PC (1, 10, and 50 μM), EA (1, 10, and 50 μM), PPPs (1, 10, and 100 μg/mL) or vehicle (0.2% DMSO) for 1 h and then were incubated with LPS (1 μg/mL) for 24 h. The amount of nitrite in the culture media was detected by NO assay kits. The absorbance at 540 nm was measured with the NaNO_2_ standard curve, and nitrite production was determined.

### Enzyme-linked immunosorbent assay

RAW264.7 macrophages were seeded in 6-well plates at a density of 4 × 10^5^ cells/well and then pretreated with the tested compounds or vehicle (0.2% DMSO) for 1 h prior to the stimulation with LPS (1 μg/mL) for 24 h. The concentrations of PGE_2_, TNF-α, IL-1β, and IL-6 released into the medium were detected by enzyme-linked immunosorbent assay (ELISA) kits (R&D Systems, Minneapolis, MN, USA) according to the manufacturer’s instructions.

### Western blot analysis

RAW264.7 macrophages (1 × 10^6^ cells/well) were plated in 6-cm dishes, incubated overnight, and were then treated with the tested polyphenols or vehicle (0.2% DMSO) for 1 h prior to LPS (1 μg/mL). Proteins of the NF-κB signaling pathway (including p65, IκB, and *p*-IκB) were collected 20 min after LPS treatment, while TLR4 protein was collected after 24 h of LPS treatment. Whole cell extracts, cytosolic and nuclear proteins were extracted, respectively, by extraction kits from Solarbio Life Sciences (Beijing, China) according to the manufacturer’s instructions. Samples (30 μg of protein) were separated on 10% SDS-PAGE and transferred to PVDF membranes (Millipore, Billerica, MA, USA). Next, the membranes were blocked with 5% nonfat milk in TBST buffer for 1 h at room temperature and then probed with primary antibodies at 4 °C overnight. The membranes were washed and incubated at room temperature for 1 h with secondary antibodies (Jackson Immuno Research, West Grove, PA, USA). Blots were detected by enhanced chemiluminescence (ECL) detection kits (Fdbio Science, China) and analyzed using Bio-1D software.

### Statistical analysis

The results were shown as mean ± SD from three independent experiments. A one-way analysis of variance (ANOVA) followed by a Dunnett’s *t*-test was applied to assess the statistical significance of the differences between the study groups (SPSS 13.0). Values with *P* < 0.05 were considered significant.

## Results

### Cytotoxicity of PPPs on RAW264.7 macrophages

Previously, we examined the cytotoxicity of PPPs, PC, and EA by MTT [3-(4,5-dimethyl-2-thiazolyl)-2,5-diphenyl-2-H-tetrazolium bromide] assay. The results showed that the cell viability was slightly affected within 100 μg/mL (PPPs), 100 μM (PC), and 50 μM (EA) for 25 h. Therefore, the concentration range of EA and PC was determined between 1 and 50 μM, and the range of 1–100 μg/mL to PPPs was adopted in the subsequent experiments.

### Inhibition of PPPs on LPS-induced TLR4 mRNA and protein expression

**Fig. 1:**
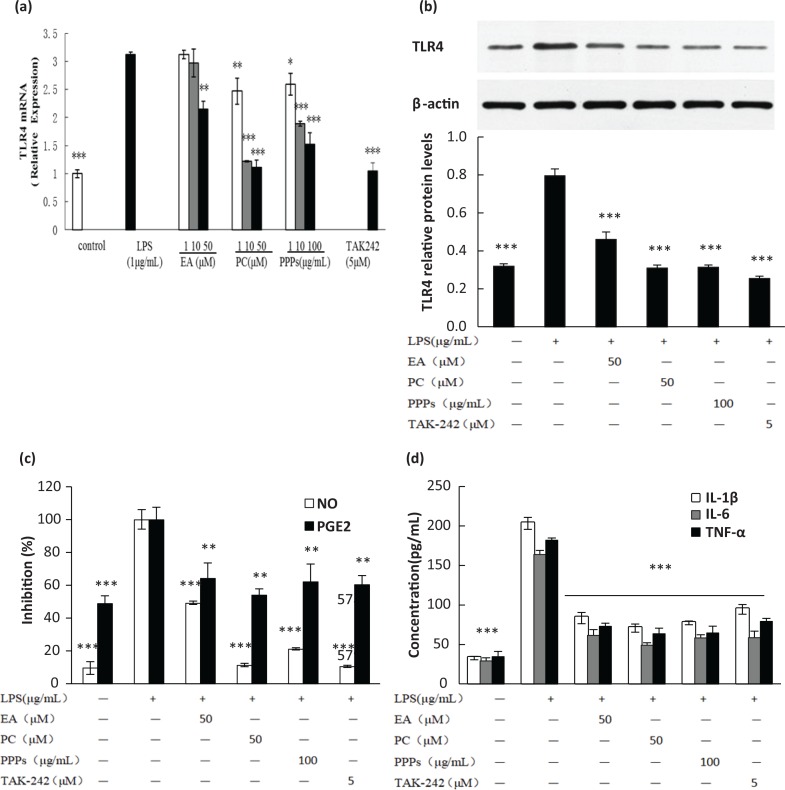
The effect of the test polyphenols on LPS-induced TLR4 mRNA and protein expression, and the related inflammatory cytokines and mediators in RAW264.7 macrophages. Cells were pretreated with different concentrations of PPPs, PC, or EA for 1 h and then stimulated with LPS (1 μg/mL) for 24 h. The total RNA and protein were extracted to perform (a) real-time PCR and (b) Western blot analysis. (c) The levels of nitrite and the concentration of PGE_2_ were measured by Griess reagents, and (d) the concentrations of TNF-α, IL-1β, and IL-6 were measured by ELISA kit in the culture media. Data are expressed as mean ± SD. **P < 0.01 and ***P < 0.001 compared with LPS-stimulated group.

To assess the inhibition of the polyphenols on the expression of TLR4, which is known as the LPS receptor, total RNA and protein were extracted to perform RT-PCR and Western blot analysis, respectively. Figure 1a depicted that LPS (1 μg/mL) stimulation for 24 h significantly increased the TLR4 mRNA expression by 3.12 times in comparison with control group. EA, PC, and PPPs pretreatment all significantly reduced LPS-induced TLR4 mRNA expression in a dose-dependent manner. EA had the lowest inhibitory effect on TLR4, which exerted significant inhibition only at 50 μM (*P* < 0.05). PC and PPPs significantly inhibited the mRNA expression of TLR4 at each concentration (*P* < 0.05). Furthermore, the inhibitory effect of PC (50 μM) was similar to that of TAK-242 (inhibitor of TLR4) and close to the control level. Moreover, the trend of TLR4 protein expression (Fig. 1b) was similar to that of the mRNA level. The tested polyphenols significantly prevented the elevated TLR4 protein expression induced by LPS (*P* < 0.001) at high concentration, and it was ranked as PC > PPPs > EA. The inhibition of the pro-inflammatory cytokines (TNF-α, IL-1β, and IL-6; Fig. 1c) and inflammatory mediators (NO and PGE_2_; Fig. 1d) by these tested polyphenols was also consistent with the results of TLR4.

### Inhibition of PPPs on intracellular ROS production

**Fig. 2:**
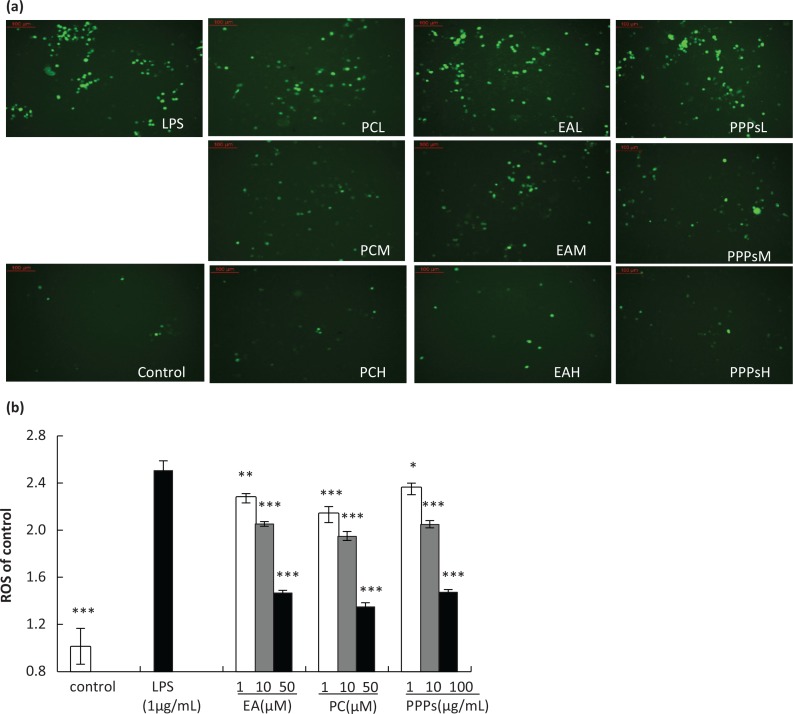
The effect of the test polyphenols on LPS-induced ROS production in RAW264.7 macrophages. Cells were pretreated with different concentrations of PPPs, PC, or EA for 1 h and then stimulated with LPS (1 μg/mL) for 24 h. The cells were incubated with 10 μM DCFH-DA for an additional 25 min. The cells were washed three times with PBS, and then (a) one part of the cells was evaluated by using a fluorescence microscope, and (b) another part of the cells was harvested and detected by fluorescence microplate reader with excitation at 488 nm and emission at 525 nm. Data are expressed as mean ± SD. *P < 0.05, **P < 0.01, and ***P < 0.001 compared with LPS-stimulated group.

The interaction of the cellular immune system with endogenous or exogenous inflammatory stimuli determines the generation of ROS, which causes the hyperactivation of inflammatory responses and leads to tissue damage and oxidative stress (29). Therefore, ROS activation could act as a significant and adverse participant in abnormal inflammatory diseases. Figure 2a and b showed the effects of the three tested polyphenols on intracellular ROS production in LPS-induced RAW264.7 macrophages. The cells stimulated with LPS (1 μg/mL) showed an intracellular ROS elevation in comparison to control group. Compared with the LPS group, EA, PC, and PPPs pretreated groups significantly reduced the levels of ROS in a dose-dependent manner. We also found that PC, EA, and PPPs in high concentration almost reversed the levels of ROS about 46.2, 41.4, and 41.2%, compared with LPS group, respectively.

### Inhibition of PPPs on NF-kB p65 activation in LPS-induced RAW264.7 macrophages

**Fig. 3:**
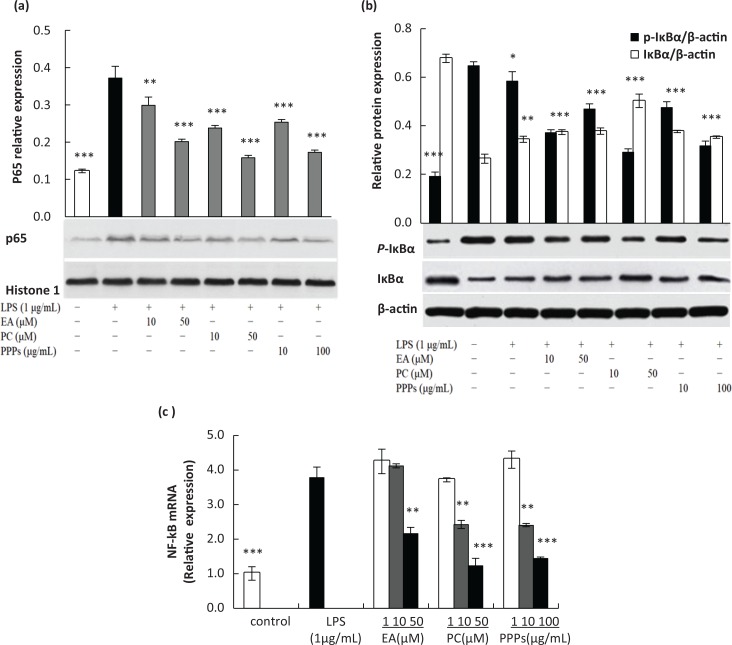
The effect of the test polyphenols on LPS-induced activation of P65, degradation and phosphorylation of IκBα, and the mRNA level of NF-κB. Cells were pretreated with different concentrations of PPPs, PC, or EA for 1 h and then stimulated with LPS (1 μg/mL) for 20 min. The nuclear P65 (a), degradation and phosphorylation of IκBα (b) were subjected by Western blot analysis. Cells were pretreated with different concentrations of PPPs, PC, or EA for 1 h and then stimulated with LPS (1 μg/mL) for 24 h. The total RNA was extracted to perform real-time PCR (c). Data are expressed as mean ± SD. *P < 0.05, **P < 0.01, and ***P < 0.001 compared with LPS group.

Accumulated evidence indicates that NF-κB is a major transcription factor that modulates the expression of COX-2, iNOS, and pro-inflammatory cytokines (TNF-α, IL-1β, and IL-6) (1). ROS is also thought to be involved in inflammatory gene expression through redox-based activation of the NF-κB signaling pathway (30). In our previous studies, we found that these three tested polyphenols had obvious inhibitory effect on these inflammatory mediators and pro-inflammatory cytokines via regulating the MAPKs pathway (28). However, it still remains unclear whether these tested polyphenols also inhibit the activation of NF-κB pathway. Figure 3a suggested that the expression of p65 in nucleus was markedly increased upon exposure to LPS alone, but PPPs, PC, and EA inhibited LPS-induced increase of nuclear p65 in a dose-dependent manner. Additionally, PC had stronger inhibition in comparison to PPPs, followed by EA. The inhibition of NF-κB mRNA level (Fig. 3c) was similar to the expression of p65 protein. PC and PPPs could significantly inhibit the NF-κB mRNA expression at middle and high concentrations (*P* < 0.01), and EA had a significant inhibitory effect at high concentration (*P* < 0.01). Figure 3b showed that the three tested polyphenols inhibited LPS-induced phosphorylation and degradation of IκBα in a dose-dependent manner, which was similar to the results of nuclear p65. Overall, these results suggested that PPPs inhibited LPS-induced p65 activation via blocking of IκBα phosphorylation and degradation.

### Effect of PPPs on LPS-induced NF-kB nuclear translocation and the productions of pro-inflammatory mediators and cytokines in RAW264.7 macrophages

**Fig. 4:**
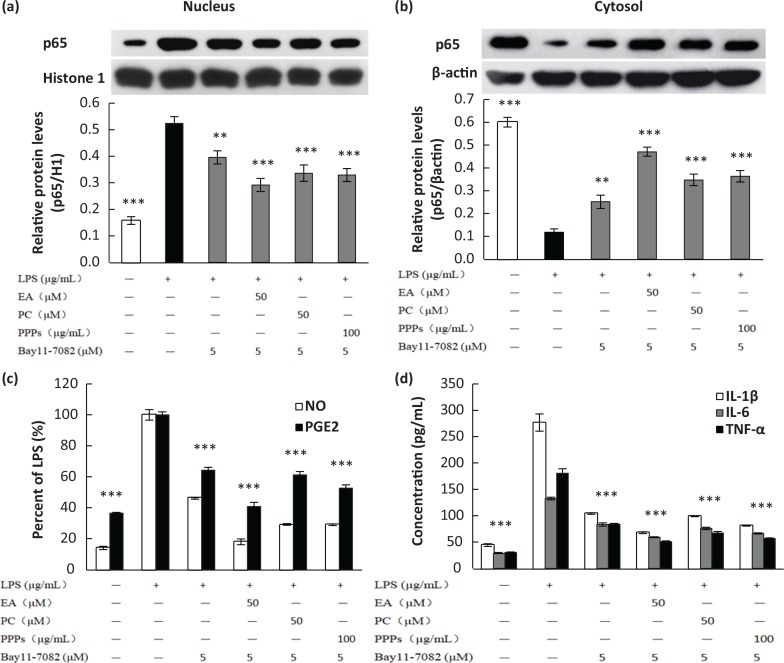
The effect of test polyphenols on LPS-induced NF-κB p65 translocation (a, b), the production of NO/PGE2 (c), and pro-inflammatory cytokines (d) in RAW264.7 macrophages. Cells were pretreated with different concentrations of PPPs, PC, EA, or Bay11-7082 (5 μM) for 1 h and then stimulated with LPS (1 μg/mL) for 20 min. The total lysates of the proteins were subjected to Western blot analysis, and the culture media were collected for NO and ELISA detections. Data are expressed as mean ± SD. **P < 0.01 and ***P < 0.001 compared with LPS-stimulated group.

Bay11-7082 is an inhibitor of IκBα phosphorylation, which can inhibit the translocation of NF-κB to the nucleus by blocking the phosphorylation of IκBα. In Fig. 4a, the nuclear protein expression of p65 is downregulated by 24.5% with Bay11-7082 pretreatment compared with the LPS-treated group, while in combination of Bay11-7082 and the tested polyphenols, the p65 expression in nucleus declined by 44.3% (PC), 30% (EA), and 33% (PPPs), respectively. The trend of p65 expression in cytosol was opposite to that of the nucleus (Fig. 4b). Moreover, the combined inhibitory effects of Bay11-7082 and the three tested polyphenols were more pronounced (*P* < 0.05) than Bay11-7082’s effect alone. So, PPPs, PC, and EA had inhibitory effects on the translocation of NF-κB to the nucleus by blocking the phosphorylation of IκBα. In parallel with the result of Western blot analysis, the combination of Bay11-7082 and the three tested polyphenols significantly prevented LPS-induced production of the pro-inflammatory cytokines (Fig. 4c and d).

### Effect of ubiquitin proteasome inhibitor and PPPs on LPS-induced IkBa-mediated degradation and NF-kB translocation in RAW264.7 macrophages

**Fig. 5:**
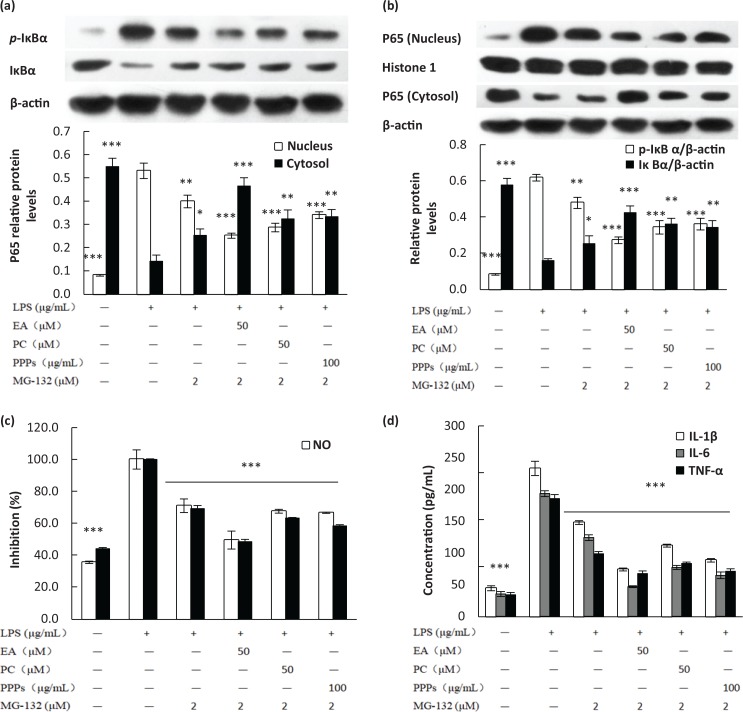
The effect of ubiquitin proteasome inhibitor on LPS-induced IκBα Ub-mediated degradation (a), NF-κB translocation (b), the production of NO/PGE_2_ (c), and pro-inflammatory cytokines (d) in RAW264.7 macrophages. Cells were pretreated with different concentrations of PPPs, PC, EA, or MG-132 (2 μM) for 1 h and then stimulated with LPS (1 μg/mL) for 20 min. The total lysates of the proteins were subjected to Western blot analysis, and the culture media were collected for NO and ELISA detections. Data are expressed as mean ± SD. *P < 0.05, **P < 0.01, and ***P < 0.001 compared with LPS-stimulated group.

NF-κB is normally sequestered in the cytoplasm by the inhibitor molecule IκB. Once activated, the translocation of NF-κB into nuclear is proceeded by the phosphorylation, ubiquitination, and degradation of IκBα (31). We had confirmed that PPPs, PC, and EA could inhibit the phosphorylation of IκBα. Therefore, we attempted to find whether these tested polyphenols could inhibit the ubiquitination and proteolytic degradation of IκBα. The results showed that the significant decrease in IκBα induced by LPS was significantly reversed by MG-132 pretreatment, an inhibitor of ubiquitin-proteasome (Fig. 5a). In addition, the combination of MG-132 and the tested polyphenols further increased the protein expression of IκBα, which indicated that the tested polyphenols had significant inhibitory effect on ubiquitination and proteolytic degradation of IκBα, like an inhibitor of ubiquitin-proteasome. The protein expression of *p*-IκBα was opposite to IκBα. Phosphorylation, ubiquitination, and proteolytic degradation of IκBα cause the release of NF-κB and its translocation from cytosol to the nucleus. MG-132 decreased nuclear protein levels of p65 and increased cytoplasmic p65 in LPS-stimulated RAW264.7 macrophages, and the inhibitory effect was further strengthened in the combination of MG-132 and the tested polyphenols (Fig. 5b). Moreover, the production of the inflammatory mediators and pro-inflammatory cytokines in the downstream of NF-κB also confirmed the above results; the combination of MG-132 and the three tested polyphenols significantly prevented LPS-induced production of the pro-inflammatory cytokines (Fig. 5c and d).

## Discussion

Inflammation is a primary physiological defense process of the biological system and protects against injuries caused by harmful stimuli such as pathogens and poisons (11). Natural products have long been recognized as an invaluable source of the most active components of medicines for treating and preventing various human diseases, including inflammatory diseases. Many studies have shown that phytochemicals such as polyphenols have anti-inflammatory effects. Pomegranate peel extracts have been focused for their richness and unique compositions in polyphenols. In our previous work, PPPs and their main polyphenol components (PC and EA) had been shown to significantly inhibit the expression of pro-inflammatory mediators and inflammatory cytokines in LPS-induced RAW264.7 macrophages. The molecular mechanism was associated with the inhibition of MAPKs activation. In the present work, we found that PPPs, PC, and EA significantly inhibited LPS-induced mRNA and protein expression of TLR4. TLRs are an integral part of the molecular mechanisms of inflammation processes. LPS activates macrophage and microglia by selectively stimulating toll-like receptor 4 (TLR4) and triggering NF-κB and MAPK pathways (32) specifically, and eventually results in the excess production of a large array of cytotoxic factors such as IL-1β, IL-6, TNF-α, NO, ROS, iNOS, and COX-2 (33, 34). This result indicated that the decreased expression of TLR4 could inhibit the initiation of intracellular signaling cascades, which subsequently suppressed the activation of NF-κB and pro-inflammatory mediators.

The generation of ROS by phagocytic leukocytes (neutrophils, monocytes, macrophages, and eosinophils) is one of the most important characteristics in the inflammatory process. Excess ROS production is detrimental to cellular functions. It is well reported that ROS caused modification of biomolecules (35). In this study, the levels of ROS were significantly increased after the LPS treatment, and PPPs PC, EA, could effectively inhibit the increase of ROS in a dose-dependent manner. ROS is thought to be involved in inflammatory gene expression through the redox-based activation of the NF-κB signaling pathway. It has been shown that many antioxidants inhibited inflammatory gene expression and NO production by suppressing NF-κB activation through the removal of ROS. Therefore, ROS may play an important role in NF-κB activation and pro-inflammatory cytokine production in LPS-induced macrophages (6).

NF-κB is a transcription factor that plays an important role in the expression of genes in immune and inflammatory responses (36). The molecular mechanisms of NF-κB activation are involved in the activation of a cascade of cytoplasmic proteins and the nuclear translocation of the NF-κB p65 subunit (37). In unstimulated cells, NF-κB exists in the cytosol in quiescent form bound to its inhibitory protein, IκB protein. In response to inflammatory stimuli, such as LPS, the IκBα inhibitory protein is phosphorylated by IκBα kinases, resulting in its ubiquitination and subsequent degradation by proteasome (38). This result leads to the release and translocation of NF-κB to the nucleus. In the nucleus, NF-κB binds to the specific DNA sequence in the promoter region of target genes and modulates their transcription (39). Therefore, we examined the effect of these tested polyphenols on NF-κB activation. Western blot results revealed that PPPs, PC, and EA inhibited LPS-induced nuclear translocation of p65. Furthermore, we found that these three tested polyphenols inhibited the phosphorylation and degradation of IκB, and the effects were stronger when co-treated with IκBα phosphorylation inhibitor (Bay11-7082) or ubiquitin proteasome inhibitor (MG-132). These results suggested that PPPs and their main components might act as the inhibitors and inhibit the activation of NF-κB by blocking LPS-induced phosphorylation and degradation of IκB and nuclear translocation of p65.

Pomegranate (*Punica granatum* L.), a nutrient-rich unique fruit, has been used for centuries for the prevention and treatment of various inflammation-driven diseases (40). Pomegranate fruits contain abundant anthocyanin and polyphenol (41), and the peel possesses the highest polyphenol content among all parts of the pomegranate. The main benefit of the pomegranate has been attributed to its unique polyphenol composition (42). Indeed, pomegranate polyphenols have been shown to exhibit high antioxidant and anti-inflammatory capacities that are of interest for the prevention of several disorders (43, 44). Shah et al. (45) reported that *P. granatum* juice and purified PC reduced NF-κB mRNA levels in colitis SD rats. Previous studies have showed the anti-inflammatory effect of pomegranate and its extract. Ramlagan et al. (46) reported that pomegranate mesocarp extract significantly reduced ROS production and downregulated the overexpression of pro-inflammatory mediators NF-κB in 3T3-L1 preadipocytes in the presence of advanced glycation end products. Romier-Crouzet et al. (47) found that pomegranate extracts could inhibit NF-κB activity in IL-1β-induced Cao-2 cells. Dell’Agli et al. (48) transfected THP-1 human monocyte leukemia cells and found that the methanolic extract of the pomegranate rind and EA and PC inhibited the hemozoin-induced NF-κB promoter activity, which indicated that they could inhibit NF-κB-driven transcription. This result was consistent with our NF-κB western blot (WB) study.

Phenolic compounds often present high antioxidant activity (49). Many viewpoints considered that polyphenols have been showed with the anti-inflammatory effect largely attributed to the strong antioxidant ability. The efficiency of ellagitannins (ETs) and EA as antioxidant compounds in pomegranate greatly depends on their chemical structure. The presence of several hydroxyl functions in ortho position in ETs is responsible for the strong ability to donate a hydrogen atom and support the unpaired electron. In addition, the antioxidant efficiency of ETs and EA is directly correlated with their degree of hydroxylation, and polyphenols with high molecular weight were the major contributors of the antioxidant capacity (50). PC has stronger antiradical activity with the stable structure, which possesses more benzene ring and phenolic hydroxyl group, compared with low molecular weight of EA. Seeram (51) reported that PC, with the stronger antioxidant activity than EA, acted as the most potent antioxidant ingredient responsible for >50% of the pomegranate juice’s antioxidative potential. Similarly, in this study, the anti-inflammatory effect of PC was better than that of EA. Winand and Schneider (52) reported that pomegranate husk extract, PC and EA, could reduce pro-inflammatory cytokines secretion, and the effect of PC was better than that of EA, which was consistent with our finding. Mo et al. (53) found that pomegranate rind extract was more active as an anti-inflammatory agent than EA in the croton oil-induced mouse ear edema, the carrageenan-induced rat paw edema, and the CFA-induced polyarthritis model. Park (54) compared the effects of PC and EA on cell viability and cell adherence of THP-1 cells and found that PC would be more useful than EA as an anti-inflammatory agent against PM10. In addition, Lee (55) isolated four hydrolysable tannins from pomegranate and found that PC had the strongest effect against acne vulgaris. Our results also demonstrated that PPPs and PC had stronger anti-inflammatory effects than EA, which indicated that PC is the most bioactive component in PPPs.

## Conclusion

This study showed that PPPs and their main components – PC and EA – could significantly inhibit LPS-induced intracellular ROS production in RAW264.7 macrophages in a dose-dependent manner. As an important receptor of LPS, the mRNA and protein expression of TLR4 was also inhibited by PPPs, PC, and EA, which is an initial step of intracellular signaling cascades in the LPS-stimulated cells. Besides the MAPK signaling pathway, the molecular mechanism of anti-inflammatory on PPPs is also associated with the NF-κB pathway. PPPs and their main components could inhibit NF-κB activation by blocking LPS-induced phosphorylation, ubiquitination, and degradation of IκB and, subsequently, could prevent p65 nuclear translocation. Therefore, PC should be carefully preserved during extraction to maintain its high anti-inflammatory effect because its hydrolytic form EA has a weaker effect. These results suggest that PPPs, a by-product of the pomegranate fruit juice industry, could be a useful and novel natural source for the prevention of inflammation-associated disorders.

## References

[1] Shao J, Li YQ, Wang ZY, Xiao MM, Yin PH, Lu YH, et al. 7b, a novel naphthalimide derivative, exhibited anti-inflammatory effects via targeted-inhibiting TAK1 following down-regulation of ERK1/2- and p38 MAPK-mediated activation of NF-κB in LPS-stimulated RAW264.7 macrophages. Int Immunopharmacol 2013; 17(2): 216–28. doi:10.1016/j.intimp.2013.06.008. 23810444

[2] Himaya SWA, Ryu BM, Qian ZJ, Kim SK Paeonol from *Hippocampus kuda* Bleeler suppressed the neuro-inflammatory responses *in vitro* via NF-κB and MAPK signaling pathways. Toxicol in Vitro 2012; 26(6): 878–87. doi:10.1016/j.tiv.2012.04.022. 22542583

[3] Park HH, Kim MJ, Li Y, Park YN, Lee J, Lee JL, et al. Britanin suppresses LPS-induced nitric oxide, PGE_2_ and cytokine production via NF-κB and MAPK inactivation in RAW 264.7 cells. Int Immunopharmacol 2013; 15(2): 296–302. doi:10.1016/j.intimp.2012.12.005. 23270759

[4] Bai SK, Lee SJ, Na HJ, Ha KS, Han JA, Lee HS, et al. β-Carotene inhibits inflammatory gene expression in lipopolysaccharide-stimulated macrophages by suppressing redox-based NF-κB activation. Exp Mol Med 2005; 37(4): 323–34. doi:10.1038/emm.2005.42. 16155409

[5] Suh SJ, Chung TW, Son MJ, Kim SH, Moon TC, Son KH, et al. The naturally occurring biflavonoid, ochnaflavone, inhibits LPS-induced iNOS expression, which is mediated by ERK1/2 via NF-κB regulation, in RAW 264.7 cells. Arch Biochem Biophys 2006; 447(2): 136–46. doi:10.1016/j.abb.2006.01.016. 16527246

[6] Kim HG, Yoon DH, Lee WH, Han SK, Shrestha B, Kim CH, et al. *Phellinus linteus* inhibits inflammatory mediators by suppressing redox-based NF-κB and MAPKs activation in lipopolysaccharide-induced RAW 264.7 macrophage. J Ethnopharmacol 2007; 114(3): 307–15. doi:10.1016/j.jep.2007.08.011. 17936530

[7] Olivera A, Moore TW, Hu F, Brown AP, Sun A, Liotta DC, et al. Inhibition of the NF-κB signaling pathway by the curcumin analog, 3, 5-Bis(2-pyridinylmethylidene)-4-piperidone (EF31): anti-inflammatory and anti-cancer properties. Int Immunopharmacol 2012; 12(2): 368–77. doi:10.1016/j.intimp.2011.12.009. 22197802 PMC3372981

[8] Blonska M, Lin X NF-κB signaling pathways regulated by CARMA family of scaffold proteins. Cell Res 2011; 21(1): 55–70. doi:10.1038/cr.2010.182. 21187856 PMC3193407

[9] Wu SJ, Chen YW, Wang CY, Shyu YT Anti-inflammatory properties of high pressure-assisted extracts of *Grifola frondosa* in lipopolysaccharide-activated RAW 264.7 macrophages. Int J Food Sci Tech 2017; 52(3): 671–8. doi:10.1111/ijfs.13320.

[10] Zhang JX, Xing JG, Wang LL, Jiang HL, Guo SL, Liu R Luteolin inhibits fibrillary β-Amyloid_1-40_-induced inflammation in a human blood-brain barrier model by suppressing the p38 MAPK-mediated NF-κB signaling pathways. Molecules 2017; 22(3): 334–53. doi:10.3390/molecules22030334. PMC6155314 28245546

[11] Shukla MSM, Gupta PDK, Rasheed PDZ, Khan PDKA, Haqqi PDTM Consumption of hydrolyzable tannins-rich pomegranate extract suppresses inflammation and joint damage in rheumatoid arthritis. Nutrition 2008; 24(7): 733–43. doi:10.1016/j.nut.2008.03.013. 18490140 PMC2577876

[12] Lee CJ, Chen LG, Liang WL, Wang CC Anti-inflammatory effects of *Punica granatum* Linne *in vitro* and *in vivo*. Food Chem 2010; 118(2): 315–22. doi:10.1016/j.foodchem.2009.04.123.

[13] Ismail T, Sestili P, Akhtar S Pomegranate peel and fruit extracts: a review of potential anti-inflammatory and anti-infective effects. J Ethnopharmacol 2012; 143(2): 397–405. doi:10.1016/j.jep.2012.07.004. 22820239

[14] Shaban NZ, El-Kersh MAL, El-Rashidy FH, Habashy NH Protective role of *Punica granatum* (pomegranate) peel and seed oil extracts on diethylnitrosamine and phenobarbital-induced hepatic injury in male rats. Food Chem 2013; 141(3): 1587–96. doi:10.1016/j.foodchem.2013.04.134. 23870864

[15] Wu D, Ma X, Tian W Pomegranate husk extract, punicalagin and ellagic acid inhibit fatty acid synthase and adipogenesis of 3T3-L1 adipocyte. J Funct Foods 2013; 5(2): 633–41. doi:10.1016/j.jff.2013.01.005.

[16] Al-Muammar MN, Khan F Obesity: the preventive role of the pomegranate (*Punica granatum*). Nutrition 2012; 28(6): 595–604. doi:10.1016/j.nut.2011.11.013. 22342388

[17] Gonzalez-Trujano ME, Pellicer F, Mena P, Moreno DA, Garcra-Viguera C Antinociceptive and anti-inflammatory activities of a pomegranate (*Punica granatum* L.) extract rich in ellagitannins. Int J Food Sci Nutr 2015; 66(4): 395–9. doi:10.3109/09637486.2015.1024208. 25822007

[18] Li JK, He XY, Li MY, Zhao W, Liu L, Kong XH Chemical fingerprint and quantitative analysis for quality control of polyphenols extracted from pomegranate peel by HPLC. Food Chem 2015; 176: 7–11. doi:10.1016/j.foodchem.2014.12.040. 25624199

[19] Lv O, Wang LF, Li JK, Ma QQ, Zhao W Effects of pomegranate peel polyphenols on lipid accumulation and cholesterol metabolic transformation in L-02 human hepatic cells *via* the PPARγ-ABCA1/CYP7A1 pathway. Food Funct 2016; 7(12): 4976–83. doi:10.1039/c6fo01261b. 27845788

[20] Zhao YH, Li JK, Li GR Purification with macroporous adsorbent resins and *in vitro* antioxidant evaluation of pomegranate peel polyphenols. Food Sci 2010; 31(11): 31–7. doi:10.7506/spkx1002-6630-201011007.

[21] Li JK, Li GX, Zhao YH, Yu CZ Composition of pomegranate peel polyphenols and its antioxidant activities. Sci Agric Sin 2009; 42(11): 4035–41. doi:10.3864/j.issn.0578-1752.2009.11.034.

[22] Song BB, Li J, Li JK Pomegranate peel extract polyphenols induced apoptosis in human hepatoma cells by mitochondrial pathway. Food Chem Toxicol 2016; 93: 158–66. doi:10.1016/j.fct.2016.04.020. 27120393

[23] Liu R, Li J, Cheng Y, Huo T, Xue J, Liu Y, et al. Effects of ellagic acid-rich extract of pomegranates peel on regulation of cholesterol metabolism and its molecular mechanism in hamsters. Food Funct 2015; 6(3): 780–7. doi:10.1039/c4fo00759j. 25579987

[24] Zhao W, Li J, He X, Lv O, Cheng Y, Liu R *In vitro* steatosis hepatic cell model to compare the lipid-lowering effects of pomegranate peel polyphenols with several other plant polyphenols as well as its related cholesterol efflux mechanisms. Toxicol Rep 2014; 1: 945–54. doi:10.1016/j.toxrep.2014.10.013. 28962306 PMC5598384

[25] Zhao SJ, Li JK, Wang LF, Wu XX Pomegranate peel polyphenols inhibit lipid accumulation and enhance cholesterol efflux in raw264.7 macrophages. Food Funct 2016; 7(7): 3201–10. doi:10.1039/c6fo00347h. 27334099

[26] Chun J, Choi RJ, Khan S, Lee DS, Kim YC, Nam YJ, et al. Alantolactone suppresses inducible nitric oxide synthase and cyclooxygenase-2 expression by down-regulating NF-κB, MAPK and AP-1 via the MyD88 signaling pathway in LPS-activated RAW 264.7 cells. Int Immumopharmacol 2012; 14(4): 375–83. doi:10.1016/j.intimp.2012.08.011. 22940184

[27] Lee HS, Ryu DS, Lee GS, Lee DS Anti-inflammatory effects of dichloromethane fraction from *Orostachys japonicas* in RAW 264.7 cells: suppression of NF-κB activation and MAPK signaling. J Ethnopharmacol 2012; 140(2): 271–6. doi:10.1016/j.jep.2012.01.016. 22285522

[28] Du L, Li J, Zhang X, Wang L, Zhang W Pomegranate peel polyphenols inhibits inflammation in LPS-induced RAW264.7 macrophages via the suppression of MAPKs activation. J Funct Foods 2018; 43: 62–9. doi:10.1016/j.jff.2018.01.028. PMC6495109 31073284

[29] Gasparrini M, Forbes-Hernandez TY, Giampieri F, Afrin S, Alvarez-Suarez JM, Mazzoni L, et al. Anti-inflammatory effect of strawberry extract against LPS-induced stress in RAW 264.7 macrophages. Food Chem Toxicol 2017; 102: 1–10. doi:10.1016/j.fct.2017.01.018. 28130090

[30] Kumar A, Wu H, Collier-Hyams LS, Hansen JM, Li T, Yamoah K, et al. Commensal bacteria modulate cullin-dependent signaling via generation of reactive oxygen species. EMBO J 2007; 26(21): 4457–66. doi:10.1038/sj.emboj.7601867. 17914462 PMC2063476

[31] Baker RG, Hayden MS, Ghosh S NF-κB, inflammation and metabolic disease. Cell Metab 2011; 13: 11–22. doi:10.1016/j.cmet.2010.12.008. 21195345 PMC3040418

[32] Fu Y, Liu B, Zhang N, Liu Z, Liang D, Li F, et al. Magnolol inhibits lipopolysaccharide-induced inflammatory response by interfering with TLR4 mediated NF-κB and MAPKs signaling pathways. J Ethnopharmacol 2013; 145(1): 193–9. doi:10.1016/j.jep.2012.10.051. 23127653

[33] Kim HS, Kim YJ, Lee HK, Ryu HS, Kim JS, Yoon MJ, et al. Activation of macrophages by polysaccharide isolated from *Paecilomyces cicadae* through toll-like receptor 4. Food Chem Toxicol 2012; 50(9): 3190–7. doi:10.1016/j.fct.2012.05.051. 22687552

[34] Wang X, Hu D, Zhang L, Lian G, Zhao S, Wang C, et al. Gomisin A inhibits lipopolysaccharide-induced inflammatory responses in N9 microglia via blocking the NF-κB/MAPKs pathway. Food Chem Toxicol 2014; 63: 119–27. doi:10.1016/j.fct.2013.10.048. 24211520

[35] Zhang H, Chen MK, Lia K, Hu C, Lu MH, Situ J Eupafolin nanoparticle improves acute renal injury induced by LPS through inhibiting ROS and inflammation. Biomed Pharmacother 2017; 85: 704–11. doi:10.1016/j.biopha.2016.11.083. 27923689

[36] Hayden MS, Ghosh S Shared principles in NF-kappaB signaling. Cell 2008; 132: 344–62. doi:10.1016/j.cell.2008.01.020. 18267068

[37] Lawrence T The nuclear factor NF-κB pathway in inflammation. CSH Perspect Biol 2009; 1(6): a001651. doi:10.1101/cshperspect.a001651. PMC2882124 20457564

[38] Xue Y, Wang Y, Feng DC, Xiao BG, Xu LY Tetrandrine suppresses lipopolysaccharide-induced microglial activation by inhibiting NF-κB pathway. Acta Pharmacol Sin 2008; 29(2): 245–51. doi:10.1111/j.1745-7254.2008.00734.x. 18215355

[39] Lo JY, Kamarudin MNA, Hamdi OAA, Awang K, Kadir HA Curcumenol isolated from *Curcuma zedoaria* suppresses Akt-mediated NF-κB activation and p38 MAPK signaling pathway in LPS-stimulated BV-2 microglial cells. Food Funct 2015; 6(11): 3550–9. doi:10.1039/c5fo00607d. 26301513

[40] Mandal A, Bhatia D, Bishayee A Anti-inflammatory mechanism involved in pomegranate- mediated prevention of breast cancer: the role of NF-κB and Nrf2 signaling pathways. Nutrients 2017; 9(5): 436. doi:10.3390/nu9050436. PMC5452166 28452959

[41] Fischer UA, Carle R, Kammerer DR Identification and quantification of phenolic compounds from pomegranate (*Punica granatum* L.) peel, mesocarp, aril and differently produced juices by HPLC-DAD–ESI/MS. Food Chem 2011; 127(2): 807–21. doi:10.1016/j.foodchem.2010.12.156. 23140740

[42] Gil MI, Tomás-Barberán FA, Hess-Pierce B, Holcroft DM, Kader AA Antioxidant activity of pomegranate juice and its relationship with phenolic composition and processing. J Agr Food Chem 2000; 48(10): 4581–9. doi:10.1021/jf000404a. 11052704

[43] Faria A, Calhau C The Bioactivity of pomegranate: impact on health and disease. Crit Rev Food Sci 2011; 51(7): 626–34. doi:10.1080/10408391003748100. 21793725

[44] Karwasra R, Kalra P, Gupta YK, Saini D, Kumar A, Singh S Antioxidant and anti-inflammatory potential of pomegranate rind extract to ameliorate cisplatin-induced acute kidney injury. Food Funct 2016; 7(7): 3091–101. doi:10.1039/c6fo00188b. 27273121

[45] Shah TA, Parikh M, Patel KV, Patel KG, Joshi CG, Gandhi GJ Evaluation of the effect of *Punica granatum* juice and punicalagin on NFκB modulation in inflammatory bowel disease. Mol Cell Biochem 2016; 419(1–2): 65–74. doi:10.1007/s11010-016-2750-x. 27352379

[46] Ramlagan P, Rondeau P, Planesse C, Neergheen-Bhujun VS, Fawdar S, Bourdon E *Punica granatum* L. mesocarp suppresses advanced glycation end products (AGEs) -and H_2_O_2_ -induced oxidative stress and pro-inflammatory biomarkers. J Funct Foods 2017; 29: 115–26. doi:10.1016/j.jff.2016.12.007.

[47] Romier-Crouzet B, Walle JVD, During A, Joly A, Rousseau C, Henry O, et al. Inhibition of inflammatory mediators by polyphenolic plant extracts in human intestinal Caco-2 cells. Food Chem Toxicol 2009; 47(6): 1221–30. doi:10.1016/j.fct.2009.02.015. 19233242

[48] Dell’Agli M, Galli GV, Bulgari M, Basilico N, Romeo S, Bhattacharya D Ellagitannins of the fruit rind of pomegranate (*Punica granatum*) antagonize *in vitro* the host inflammatory response mechanisms involved in the onset of malaria. Malaria J 2010; 9(1): 208. doi:10.1186/1475-2875-9-208. PMC2912927 20642847

[49] Xiang J, Apea-Bah FB, Ndolo VU, Katundu MC, Beta T Profile of phenolic compounds and antioxidant activity of finger millet varieties. Food Chem 2019; 275: 361–8. doi:10.1016/j.foodchem.2018.09.120. 30724208

[50] Landete JM Ellagitannins, ellagic acid and their derived metabolites: a review about source, metabolism, functions and health. Food Res Int 2011; 44(5): 1150–60. doi:10.1016/j.foodres.2011.04.027.

[51] Seeram NP, Adams LS, Henning SM, Niu Y, Zhang Y, Nair MG, et al. In vitro antiproliferative, apoptotic and antioxidant activities of punicalagin, ellagic acid and a total pomegranate tannin extract are enhanced in combination with other polyphenols as found in pomegranate juice. J Nutr Biochem 2005; 16(6): 360–7. doi:10.1016/j.jnutbio.2005.01.006. 15936648

[52] Winand J, Schneider YJ The anti-inflammatory effect of a pomegranate husk extract on inflamed adipocytes and macrophages cultivated independently, but not on the inflammatory vicious cycle between adipocytes and macrophages. Food Funct 2014; 5(2): 310–8. doi:10.1039/c3fo60443h. 24336779

[53] Mo J, Panichayupakaranant P, Kaewnopparat N, Nitiruangjaras A, Reanmongkol W Topical anti-inflammatory and analgesic activities of standardized pomegranate rind extract in comparison with its marker compound ellagic acid *in vivo*. J Ethnopharmacol 2013; 148(3): 901–8. doi:10.1016/j.jep.2013.05.040. 23743057

[54] Park S, Seok JK, Kwak JY, Suh HJ, Kim YM, Boo YC Anti-inflammatory effects of pomegranate peel extract in THP-1 cells exposed to particulate matter PM10. Evid Based Compl Alternat Med 2016; 51(3): 469–78. doi:10.1155/2016/6836080. PMC4877489 27247608

[55] Lee CJ, Chen LG, Liang WL, Wang CC Multiple activities of *Punica granatum* Linne against acne vulgaris. Int J Mol Sci 2017; 18(1): 141–52. doi:10.3390/ijms18010141. PMC5297774 28085116

